# Gait characteristics and factors associated with fall risk in patients with dementia with Lewy bodies

**DOI:** 10.3389/fneur.2025.1670016

**Published:** 2025-09-24

**Authors:** Zhou Su, Mengran Liu, Jun Kuai, Tingting Yi, Yuechang Zheng, Congcong Wang, Junyu Peng, Xiaojun Tian

**Affiliations:** ^1^Department of Neurology, The First Affiliated Hospital of Xinxiang Medical University, Xinxiang, Henan, China; ^2^School of Education, University of Bristol, Bristol, United Kingdom; ^3^Department of Gastroenterology, The First Affiliated Hospital of Xinxiang Medical University, Xinxiang, Henan, China

**Keywords:** dementia with Lewy bodies, gait analysis, cognitive function, fall risk, gait parameters

## Abstract

**Objective:**

To investigate the association between gait parameters and cognitive decline in patients with dementia with Lewy bodies (DLB) and evaluate the impact of gait abnormalities on fall risk.

**Methods:**

This cross-sectional study enrolled 63 DLB patients. Gait analysis, including gait speed, stride length, gait symmetry, and swing time, was performed using a pressure-sensing walkway. Cognitive function was assessed using the MoCA and MMSE. Spearman correlation analysis and multiple linear regression models were used to examine the relationship between gait parameters and cognitive function. Logistic regression models, adjusted for potential confounders, were employed to analyze the effect of gait abnormalities on fall risk.

**Results:**

Gait speed showed significant positive correlations with MoCA score (*p* = 0.001) and stride length (*p* = 0.003), and a positive correlation with MMSE score (*p* = 0.005). Gait symmetry was weakly positively correlated with MMSE score (*p* = 0.027). Patients with MoCA scores below 20 exhibited a 22% reduction in gait speed (*p* = 0.002), shortened stride length (*p* = 0.001), decreased gait symmetry (*p* = 0.034), and prolonged swing time (*p* = 0.021) compared to those with higher scores. Logistic regression analysis revealed that for each 1 standard deviation decrease in gait speed, fall risk increased by 33% (*p* = 0.001). For each 1-cm decrease in stride length, fall risk increased by 21% (*p* = 0.025). For each 1-unit decrease in gait symmetry, fall risk increased by 28% (*p* = 0.007). Patients with a history of falls demonstrated more pronounced gait deterioration. Specifically, patients with more than 2 falls exhibited a 13% reduction in stride length (*p* = 0.011) and a 12% prolongation in swing time (*p* = 0.022).

**Conclusion:**

Gait abnormalities are associated with cognitive decline, and reduced gait speed and gait asymmetry are markers of cognitive decline and increased fall risk in patients with established DLB.

## 1 Introduction

Dementia with Lewy bodies (DLB) is the second most prevalent neurodegenerative dementia after Alzheimer’s disease (AD) (1), characterized by heterogeneous clinical features encompassing cognitive decline, motor disturbances, and neuropsychiatric symptoms (2), with 68% experiencing recurrent falls causing fractures (35%), hospitalization (28%), or triple mortality risk versus non-fallers (3, 4). In recent years, falls among DLB patients have garnered increasing clinical attention due to their profound impact on autonomy and significant association with severe injury and mortality (5).

Gait control represents a highly integrated neural network process. Concurrent pathological damage across multiple nodes of this network results in pervasive gait abnormalities (6). In AD, reduced gait speed and increased stride time variability correlate with executive and memory deficits, tied to atrophy in the inferior parietal lobule and middle temporal gyrus. In Parkinson’s disease (PD), reduced stride length, gait variability, and freezing of gait reflect attentional and executive dysfunction due to basal ganglia and frontal cortex pathology. These findings highlight gait as a marker of cognitive decline in AD and PD. However, DLB-specific gait-cognition relationships, despite unique neuropathology such as *α*-synuclein and beta-amyloid, remain underexplored, with preliminary evidence suggesting distinct gait patterns like high stride-length variability (7, 8). The motor-cognitive impairment in DLB fundamentally reflects widespread pathology within the cortico-basal ganglia-cerebellar circuitry, drives accelerated functional decline, and impairs postural control (9–11).

Preliminary evidences suggest patients exhibit unique spatiotemporal gait patterns (e.g., high stride-length variability during dual-tasking), but quantitative validation is lacking (12, 13). Crucially, no studies have concurrently modeled gait parameters, cognitive trajectories, and fall risk in DLB using instrumented analysis—a gap limiting early intervention. To address these gaps, we employed GAITRite^®^ instrumented walkway analysis—a gold standard for quantifying spatiotemporal parameters (speed, stride length, symmetry)—paired with standardized cognitive assessment (MoCA, MMSE) and fall tracking. The objective of this study was to investigate the association between gait parameters and cognitive decline in patients with DLB and to evaluate the impact of gait abnormalities on fall risk. This approach overcomes subjectivity in clinical scales while capturing multidimensional gait-cognition interactions. While our study focuses on patients with confirmed DLB, gait analysis may hold potential for early detection and intervention in future studies involving prodromal DLB populations. In this study, we hypothesized that in DLB patients, specific gait parameters would be significantly associated with cognitive decline, as measured by MoCA and MMSE scores. These gait abnormalities would independently predict increased fall risk, reflecting underlying motor-cognitive network dysfunction.

## 2 Methods

### 2.1 Subject characteristics

This cross-sectional observational study enrolled 63 patients diagnosed with DLB at the Department of Neurology outpatient and inpatient units of our hospital between January 2020 and June 2024. The mean age of the patients was 62.8 ± 6.5 years. Among the patients, 30 (47.6%) were male. Cognitive function was assessed using the Montreal Cognitive Assessment (MoCA, mean score 19.6 ± 4.3) and Mini-Mental State Examination (MMSE, mean score 22.8 ± 3.7). Demographic and clinical characteristics of the patients are summarized in Table 1. DLB patients were diagnosed according to the fourth criteria for the diagnosis and management of dementia with Lewy bodies (2). These patients were initially present with at least two core clinical features of DLB (fluctuating cognition, visual hallucinations, parkinsonism, and/or rapid eye movement sleep behavior disorder) or one core clinical feature with at least one indicative biomarker including reduced dopamine transporter uptake in the basal ganglia demonstrated by single-photon emission computed tomography (SPECT) or positron emission tomography-computed tomography (PET), abnormal (low uptake) 123-Iodine-MIBG myocardial scintigraphy, and RBD screening questionnaire (RBD-SQ) and/or polysomnographic confirmation of RBD. The enrolled patients showed relative preservation of medial temporal lobe structures on MRI and/or CT. All clinical diagnoses of dementia were confirmed by consensus agreement of at least two experienced neurologists, following a case review according to the protocol. Patients meeting any of the following exclusion criteria were excluded from this study: (1) patients with other severe neurodegenerative disorders, such as Alzheimer’s disease, Parkinson’s disease, or Huntington’s disease; (2) patients with severe motor impairment precluding independent walking or participation in gait analysis; (3) patients with a history of stroke, major surgery, or other serious illness significantly affecting gait within the past 6 months; (4) patients with profound cognitive impairment rendering them unable to cooperate with cognitive testing; (5) patients or their legal representatives refusing to participate or withdrawing informed consent. This study was approved by the Medical Ethics Committee of our hospital (Approval No.: [EC-024-523]). Informed consent was obtained from all participants or their legal guardians in accordance with the ethical principles of the Helsinki Declaration.

**Table 1:** Demographic and clinical characteristics of the DLB participants.

Characteristics	Total (*n* = 63)
Age, years	62.8 ± 6.5
Sex (male *n*, %)	30 (47.6%)
Disease duration, years	4.5 ± 1.8
Hypertension, yes (*n*, %)	21 (33.3%)
Diabetes mellitus, yes (*n*, %)	13 (20.6%)
Heart disease, yes (*n*, %)	10 (15.9%)
Use of anti-parkinsonism medication, yes (*n*, %)	29 (46.0%)
History of falls (past 6 months)	43 (68.3%)
Recurrent falls (≥2 falls)	15 (23.8%)
MoCA score	19.6 ± 4.3
MMSE score	22.8 ± 3.7

DLB, Dementia with Lewy bodies; MMSE, Mini-Mental State Examination; MoCA, Montreal Cognitive Assessment.

### 2.2 Data collection of cognitive function, gait and fall risk assessment

Cognitive function was assessed by trained neuropsychological assessors using the Montreal Cognitive Assessment (MoCA) (14) and Mini-Mental State Examination (MMSE) (15).

Gait assessment was performed using the GAITRite^®^ system, a pressure-sensitive walkway, to precisely record gait parameters (16). Patients wore comfortable shoes and walked an 8-meter distance on the walkway, completing three round trips. The average value from the three trials was used for analysis. The system automatically recorded the following gait parameters: Gait Speed (m/s): Reflecting overall motor function and serving as a key indicator of mobility (17). Stride Length (cm): The distance covered in a single stride, indicative of gait regularity and step stability (18). Gait Symmetry: Quantified using the Symmetry Index (SI), calculated based on the difference in step length between the left and right feet. A value closer to 1 indicates better symmetry (19). Swing Time (s): The duration of the swing phase for each step, reflecting gait fluidity and balance control (20).

Fall Risk Assessment: Fall History Questionnaire: Patients or their caregivers retrospectively reported fall incidents occurring within the past 6 months. Details recorded included frequency, timing, circumstances, and severity of falls. Based on the number of falls, patients were categorized into: “No Falls” (0 falls), “Occasional Falls” (1–2 falls), and “Recurrent Falls” (≥3 falls). Tinetti Balance and Gait Assessment: This standardized scale, with a maximum total score of 28 points, comprises two subsections: Gait (score range: 0–12) and Balance (score range: 0–16). Lower total scores indicate a higher risk of falls.

### 2.3 Statistical analyses

All statistical analyses and data management were performed using SPSS 26.0 for Mac (IBM Corporation, Armonk, NY, United States). Continuous variables were expressed as mean ± standard deviation (SD) when normally distributed or as median (interquartile range) for non-normally distributed data. Categorical variables were summarized as frequencies (n) with percentages (%) and analyzed using *χ*^2^ test as appropriate. Spearman correlation analysis was performed to examine the association between gait parameters and cognitive function scores. Multivariable logistic regression analysis was employed to evaluate the impact of gait abnormalities on fall risk, adjusting for potential confounding factors. One-way analysis of variance (ANOVA) was performed to comparisons among groups involving normally distributed data. *Post hoc* pairwise comparisons were conducted using either Tukey’s Honestly Significant Difference (HSD) test or the Bonferroni correction for multiple comparisons. For comparisons among groups involving non-normally distributed data, the Kruskal–Wallis *H* test was used. Post-hoc pairwise comparisons were subsequently performed using Dunn’s test with appropriate adjustment for multiple comparisons. All *p*-values reported are two-tailed, and *p* < 0.05 was considered statistically significant.

To ensure our study was adequately powered to detect clinically meaningful associations, *a priori* power calculations were performed based on expected effect sizes for key outcomes. For the multivariable logistic regression model (predicting fall risk), with an observed odds ratio of 1.33 for gait speed and a baseline fall prevalence of 68.3% (based on our data, 43/63 (68.3%) of participants had a history of falls), the analysis achieved 87% power (at *α* = 0.05), well above the conventional 80% threshold. For the one-way ANOVA (comparing gait parameters across fall-frequency groups), with an effect size of *f* = 0.41, the analysis achieved 93% power at α = 0.05. These results demonstrate that our sample size (*n* = 63) was sufficient to detect the clinically relevant effects.

The associations between gait parameters and cognitive function (MoCA/MMSE scores) were first explored using unadjusted Spearman rank correlation analysis. To control for potential confounders and to ensure methodological consistency with the fall risk analysis, these associations were further assessed using multiple linear regression models, adjusting for the same covariates employed in the logistic regression models (i.e., age, sex, DLB disease duration). The variance inflation factor (VIF) was examined for all models to confirm the absence of multicollinearity (all VIF < 2.0).

## 3 Results

Gait parameters were analyzed for 63 DLB patients. Mean gait parameters were as follows: gait speed, 0.92 ± 0.18 m/s; stride length, 56.7 ± 7.5 cm; gait symmetry index, 0.88 ± 0.09; and swing time, 1.02 ± 0.15 s. Detailed gait characteristics of the patients are presented in Table 2.

**Table 2:** Gait characteristics of the DLB participants.

Characteristics	Total (*n* = 63)
Gait speed, m/s	0.92 ± 0.18
Stride length, cm	56.7 ± 7.5
Gait symmetry index	0.88 ± 0.09
Swing time, s	1.02 ± 0.15

DLB, Dementia with Lewy bodies.

Association analysis revealed significant associations between specific gait parameters and cognitive function scores (Table 3). Gait speed demonstrated significant positive correlations with both MoCA score (*r* = 0.48, *p* = 0.001) and MMSE score (*r* = 0.35, *p* = 0.005). Stride length also showed significant positive correlations with MoCA score (*r* = 0.42, *p* = 0.003) and MMSE score (*r* = 0.33, *p* = 0.017). A weak positive correlation was observed between gait symmetry and MMSE score (*r* = 0.30, *p* = 0.027). After adjusting for age, sex, and disease duration, multiple linear regression models confirmed that slower gait speed and shorter stride length remained significantly associated with lower MoCA (*β* = 0.43, 95% CI: 0.15 to 0.71, *p* = 0.003; *β* = 0.38, 95% CI: 0.10 to 0.66, *p* = 0.009) and MMSE (*β* = 0.31, 95% CI: 0.03 to 0.59, *p* = 0.031; *β* = 0.28, 95% CI: 0.00 to 0.56, *p* = 0.049) scores. The association between gait symmetry and MMSE score was attenuated but trended toward significance (*β* = 0.25, 95% CI: −0.03 to 0.53, *p* = 0.082).

**Table 3:** Association between gait parameters and cognitive function scores in DLB patients.

Gait parameter	Unadjusted analysis		Adjusted analysis (linear regression)		
	Spearman’s *ρ*	*p*-value	*β* (95% CI)	*P*-value	Adjusted *R*^2^
MoCA score					
Gait speed (m/s)	0.48	0.001	0.43 (0.15 to 0.71)	0.003	0.32
Stride length (cm)	0.42	0.003	0.38 (0.10 to 0.66)	0.009	0.28
Gait symmetry index	0.29	0.034	0.18 (−0.10 to 0.46)	0.205	0.12
Swing time (s)	−0.21	0.073	−0.15 (−0.43 to 0.13)	0.291	0.09
MMSE score					
Gait speed (m/s)	0.35	0.005	0.31 (0.03 to 0.59)	0.031	0.22
Stride length (cm)	0.33	0.017	0.28 (0.00 to 0.56)	0.049	0.19
Gait symmetry index	0.30	0.027	0.25 (−0.03 to 0.53)	0.082	0.16
Swing time (s)	−0.18	0.082	−0.12 (−0.40 to 0.16)	0.391	0.07

DLB, Dementia with Lewy bodies; MoCA, Montreal Cognitive Assessment; MMSE, Mini-Mental State Examination; *P* < 0.05 significant difference.

Adjusted for age, sex, DLB disease duration; β represents the change in cognitive score per unit increase in the gait parameter; CI, confidence interval.

Patients exhibiting slower gait speed had significantly worse cognitive function. Notably, those with MoCA scores below 20 displayed pronounced gait impairments compared to patients with higher scores (Table 4). This group exhibited a mean 22% reduction in gait speed (*p* = 0.002), significantly shortened stride length (*p* = 0.001), decreased gait symmetry (*p* = 0.034), and prolonged swing time (*p* = 0.021).

**Table 4:** Comparison of gait parameters between DLB patients grouped by MoCA score.

Gait parameter	MoCA ≥20 (*n* = 32)	MoCA <20 (*n* = 31)	*t*	*P*-value
Gait speed (m/s)	0.95 ± 0.16	0.74 ± 0.12	3.291	0.002
Stride length (cm)	59.8 ± 6.7	47.5 ± 5.3	4.017	0.001
Gait symmetry index	0.91 ± 0.08	0.78 ± 0.07	2.152	0.034
Swing time (s)	0.96 ± 0.12	1.10 ± 0.14	2.323	0.021

DLB, Dementia with Lewy bodies; MoCA, Montreal Cognitive Assessment; *P* < 0.05 significant difference.

**Figure 1:**
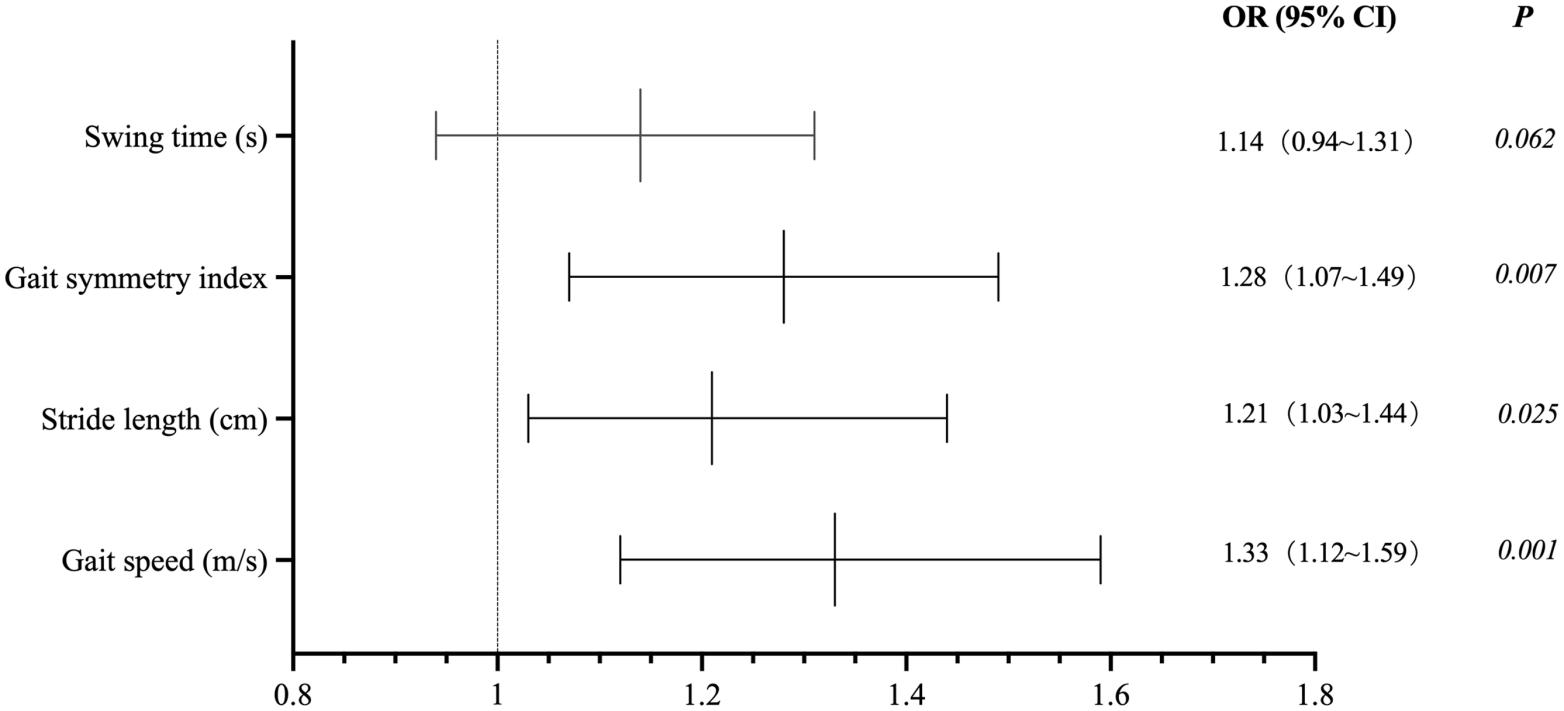
Logistic regression analysis of fall risk predictors in DLB patients. Multivariable logistic regression analysis, adjusted for age, sex, DLB disease duration, and cognitive scores, identified specific gait abnormalities as significant independent predictors of increased fall risk.

Multivariable logistic regression analysis, adjusted for age, sex, DLB disease duration, and cognitive scores, identified specific gait abnormalities as significant independent predictors of increased fall risk (Figure 1). For each 1-standard deviation (SD) decrease in gait speed, fall risk increased by 33% (OR = 1.33, 95% CI: 1.12–1.59, *p* = 0.001). Each 1-cm decrease in stride length was associated with a 21% increase in fall risk (OR = 1.21, 95% CI: 1.03–1.44, *p* = 0.025). Furthermore, each 1-unit decrease in gait symmetry corresponded to a 28% increase in fall risk (OR = 1.28, 95% CI: 1.07–1.49, *p* = 0.007).

Analysis of fall frequency confirmed that gait deterioration was more severe in patients with a history of falls. Specifically, patients experiencing recurrent falls (≥ 2 falls) showed significant gait abnormalities compared to those with no falls (Tables 5, 6): a 13% reduction in stride length (*p* = 0.011) and a 12% prolongation in swing time (*p* = 0.022).

**Table 5:** Gait parameter changes by fall frequency groups in DLB patients.

Fall frequency	Gait speed (m/s)	Stride length (cm)	Swing time (s)
No falls	0.96 ± 0.17	58.5 ± 6.9	0.98 ± 0.14
Occasional falls (1–2 falls)	0.90 ± 0.19	55.3 ± 7.2	1.05 ± 0.16
Recurrent falls (≥3 falls)	0.84 ± 0.18	51.3 ± 6.5	1.10 ± 0.18
ANOVA	4.219	5.132	4.357
*P-*value	0.021	0.014	0.021

DLB, Dementia with Lewy bodies; *p* < 0.05 significant difference.

**Table 6:** *Post hoc* pairwise comparisons of gait parameters by fall frequency groups.

Gait parameter	Comparison	Tukey *q*-statistic	*P*-value	Partial *η*^2^	Cohen’s *d*
Gait speed (m/s)	No falls vs. Occasional falls	3.02	0.062	0.047	0.62
	No falls vs. Recurrent falls	4.21	0.018	0.091	0.95
	Occasional vs. Recurrent falls	3.67	0.048	0.069	0.76
Stride length (cm)	No falls vs. Occasional falls	4.11	0.045	0.086	0.89
	No falls vs. Recurrent falls	5.13	0.011	0.134	1.24
	Occasional vs. Recurrent falls	3.97	0.032	0.081	0.84
Swing time (s)	No falls vs. Occasional falls	3.98	0.053	0.080	0.83
	No falls vs. Recurrent falls	4.35	0.022	0.095	0.98
	Occasional vs. Recurrent falls	3.87	0.041	0.075	0.80

*P* < 0.05 significant difference.

As shown in Table 7, the correlation between gait speed and MoCA score was strong (Cohen’s *d* = 1.12). One-way ANOVA comparing gait parameters across fall-frequency groups (no falls, 1–2 falls, ≥3 falls) revealed significant differences in stride length (*F* = 5.132, *p* = 0.014, partial *η*^2^ = 0.146), indicating a large effect size. *Post hoc* Tukey’s HSD tests showed that patients with recurrent falls (≥3) had a 13% reduction in stride length compared to the no-fall group (*p* = 0.011, Cohen’s *d* = 1.24) and a 12% prolongation in swing time (*p* = 0.022, Cohen’s *d* = 0.98). Multivariable logistic regression identified gait speed (OR = 1.33 per 1-SD decrease, *p* = 0.001) and stride length (OR = 1.21 per 1-cm decrease, *p* = 0.025) as significant predictors of fall risk.

**Table 7:** Effect sizes for key study findings.

Analysis method	Comparison/predictor	Statistical result	Effect size metric	Effect size value	Interpretation
Spearman correlation	Gait speed vs. MoCA score	*r* = 0.48, *p* = 0.001	Cohen’s *d*	*d* = 1.12	Large
Spearman correlation	Stride length vs. MoCA score	*r* = 0.42, *p* = 0.003	Cohen’s *d*	*d* = 0.93	Large
Logistic regression	Gait speed (per 1 SD ↓)	OR = 1.33, *p* = 0.001	Odds ratio	1.33	Significant
Logistic regression	Stride Length (per 1 cm ↓)	OR = 1.21, *p* = 0.025	Odds ratio	1.21	Significant
One-way ANOVA	Stride length (between groups)	*F* = 5.132, *p* = 0.014	Partial eta squared (η^2^)	*η*^2^ = 0.146	Large
*Post hoc* (Tukey)	No falls vs. ≥3 falls (stride length)	*p* = 0.011	Cohen’s *d*	*d* = 1.24	Large
*Post hoc* (Tukey)	No falls vs. ≥3 falls (swing time)	*p* = 0.022	Cohen’s *d*	d = 0.98	Large

Effect size interpretation: Cohen’s *d*: 0.2 (small), 0.5 (medium), 0.8 (large); Partial *η*^2^: 0.01 (small), 0.06 (medium), 0.14 (large). Odds Ratio (OR) > 1 indicates increased risk; OR = 1.33 corresponds to a 33% increased risk.

## 4 Discussion

Our study hypothesized that specific gait parameters in patients with DLB would exhibit a significant association with cognitive decline and predict an increased risk of falls, thereby reflecting dysfunction within the motor-cognitive network. The findings of our study largely supported this hypothesis. Reduced gait speed and stride length strongly correlated with lower MoCA scores. Critically, gait abnormalities independently predicted fall risk: each 1-SD decrease in speed increased falls by 33%, while a 1-cm stride reduction raised risk by 21%. An association emerged between fall frequency and gait decline: recurrent fallers showed 13% shorter stride length and 12% longer swing time versus non-fallers. These findings position gait analysis as a dual biomarker for cognitive and motor risk stratification in DLB.

The association between reduced gait velocity and executive dysfunction identified in this study aligns with recent findings in AD (21). The study has demonstrated that slower gait velocity correlates significantly with atrophy in the inferior parietal lobule, middle temporal gyrus, and insular cortex during the AD prodromal stage (21, 22). This relationship may be particularly pronounced in DLB due to its characteristic dual pathological burden of both *α*-synuclein and beta-amyloid (10). Aberrant α-synuclein deposition not only disrupts motor regulation in the basal ganglia but also spreads via limbic pathways to the prefrontal cortex, impairing executive functions. This dual pathology likely underlies the stronger gait-cognition association observed in DLB patients compared to those with pure AD or PD (10, 11).

Gait control represents a highly integrated neural network process (9). Reduced gait symmetry may directly reflect asymmetric dopaminergic degeneration within the basal ganglia—a pathological feature similar to PD but more extensive in DLB (23). Recent neuroimaging evidence reveals significantly weakened frontal-basal ganglia functional connectivity during walking tasks in DLB patients, providing direct support for a shared neural substrate underlying both executive dysfunction and gait slowing (24).

This study found that DLB patients with MoCA scores <20 exhibited a 22% reduction in gait velocity—a significantly greater decline than the approximately 15% observed in prodromal AD patients (25). This disparity may stem from DLB’s characteristic widespread neurotransmitter deficits (involving dopamine, acetylcholine, and other systems), driving simultaneous rapid deterioration of motor and cognitive functions (10, 11). Gait parameters, particularly stride time variability during dual-task walking, may serve as sensitive indicators for monitoring cognitive decline in DLB (26).

Fall risk in DLB arises from multilevel neurological dysfunction. Concurrent damage to these structures (such as basal ganglia, brainstem reticular formations, cerebellum, and prefrontal cortex.) impairs postural adjustments (3–5). In our study, multivariate logistic regression analysis demonstrated that gait speed, step length, and gait symmetry index were significant predictors of fall risk. Nevertheless, further cross-validation or external validation is required to assess the generalizability of the predictive performance of these gait parameters. Notably, reduced stride length emerged as an independent fall risk factor—a seemingly paradoxical finding that reflects compensatory adaptation (27). When sensing impaired balance, patients instinctively adopt a cautious gait strategy: shortening steps, slowing speed, and increasing double-limb support time (5). However, this fear-driven adaptation disrupts natural gait rhythm, further impairing coordination (28). This fundamentally differs from PD’s festinating gait, where primary motor control failure occurs (10, 11, 29).

The association between gait parameters and cognitive function suggests that gait abnormalities in DLB may reflect underlying disruptions in the motor-cognitive network. This relationship is clinically significant in understanding fall risk, as cognitive impairments can exacerbate gait disturbances, such as reduced gait speed and shortened stride length, thereby increasing the likelihood of postural instability and falls (30, 31). Moreover, after adjusting for multiple confounding variables, the same gait features—particularly decreased speed and stride length—were found to be independently linked to both poorer cognitive performance and a higher risk of falls. These findings suggest that a shared neuropathophysiological mechanism may exist, potentially involving dysfunction of the corticostriatal pathway, which manifests as gait deterioration. Such gait changes not only serve as indicators of brain health but also directly contribute to adverse functional outcomes, including falls. Logistic regression analyses further confirmed that gait speed, stride length, and gait symmetry independently predict fall risk, highlighting their clinical relevance. In summary, these findings support the use of gait parameters as potential biomarkers for cognitive decline and fall risk in DLB, reflecting the disease’s broader pathological processes and underscoring their utility in clinical risk stratification and intervention planning.

Gait deterioration and falls form a self-reinforcing cycle. Patients with prior falls exhibited a 13% reduction in stride length and 12% prolongation of swing time, with greater severity following recurrent falls. This represents the neuromotor manifestation of post-fall syndrome: diminished activity confidence, disuse muscle atrophy, and vestibular dysfunction collectively worsen gait control (27). DLB patients’ impaired visuospatial function and hallucinations markedly reduce environmental hazard awareness, increasing fall risk in complex settings (30, 31). Compared to AD, gait slowing emerges earlier in DLB and correlates more strongly with executive dysfunction (32, 33). Unlike PD’s characteristic “freezing of gait,” DLB typically presents with global gait parameter deterioration (34–36). Temporally, DLB exhibits a biphasic deterioration trajectory in gait-cognition relationships: gait slowing appears early alongside attentional/executive decline; subsequently, gait symmetry and variability worsen with progressive basal ganglia and posterior cortical involvement (37, 38). This pattern suggests gait metrics could serve as DLB staging biomarkers.

Although DLB shares pathological features with both PD and AD, the evidence on whether it exhibits a distinct gait pattern remains conflicting, limiting the utility of gait analysis for differential diagnosis. While some studies find no significant differences in spatiotemporal parameters such as reduced velocity and decreased stride length between DLB and AD (32), recent research suggests DLB exhibits greater gait variability, asymmetry, and postural control deficits, reflecting executive and visuospatial impairments (33). Compared to PD dementia, DLB gait overlaps significantly, with heightened asymmetry distinguishing it from AD but not reliably from PD (39). Future studies combining gait analysis with neuroimaging and biomarkers are needed to clarify DLB-specific gait signatures.

This present study has several limitations. First, its cross-sectional design inherently precludes causal inferences regarding the temporal sequence between gait deterioration and cognitive decline. Second, a modest sample size from a single center limits generalizability to broader DLB populations. Third, the absence of neuroimaging correlates hinders direct verification of neural circuit disruptions underlying gait-cognitive associations. Fourth, inadequate control for medication effects—particularly acute dopaminergic impacts of anti-Parkinsonian agents—may confound long-term gait trajectory analyses. Future studies should implement longitudinal cohort designs with biannual gait-cognitive assessments over ≥3 years to establish temporal dynamics, integrate multimodal neuroimaging (e.g., DTI for white matter integrity; resting-state fMRI for network connectivity) to map neural substrates of gait-cognition coupling, and deploy wearable inertial sensors for continuous home-based monitoring, capturing real-world fall risk factors during daily activities. Fifth, our primary analysis used absolute measures for gait symmetry and swing time without normalizing for individual step length or gait cycle duration, respectively. This may limit the clinical interpretability of these parameters, as the significance of absolute differences in step length or swing time varies with overall gait characteristics. Future research should prioritize longitudinal studies incorporating normalized gait metrics, such as step length–adjusted gait symmetry and swing time as a percentage of the gait cycle, to account for inter-individual variability in gait characteristics. Finally, incorporating dual-task walking paradigms could further elucidate cognitive-motor interactions in DLB, enhancing the sensitivity of gait as a biomarker for cognitive decline and fall risk.

## 5 Conclusion

Gait parameters, especially speed, stride length, and symmetry, serve as sensitive biomarkers for cognitive decline and fall risk in DLB. Their disruption reflects diffuse pathology across motor-cognitive networks. Clinical implementation of gait assessment can stratify risk and guide targeted interventions. Future research must validate these metrics in longitudinal cohorts and integrate them with digital health technologies for proactive DLB management.

## Data Availability

The raw data supporting the conclusions of this article will be made available by the authors, without undue reservation.
